# Twelve-Lead ECG, Holter Monitoring Parameters, and Genetic Testing in Brugada Syndrome: Insights from Analysis of Multigenerational Family with a History of Sudden Cardiac Arrest during Physical Activity

**DOI:** 10.3390/jcm12206581

**Published:** 2023-10-18

**Authors:** Paweł T. Matusik, Piotr Bijak, Magdalena Kaźnica-Wiatr, Marek Karpiński, Patrycja S. Matusik, Andrzej Maziarz, Piotr Podolec, Jacek Lelakowski

**Affiliations:** 1 Institute of Cardiology, Faculty of Medicine, Jagiellonian University Medical College, 31-202 Kraków, Poland; 2 Department of Electrocardiology, The John Paul II Hospital, 31-202 Kraków, Poland; 3 Cardiology Outpatient Clinic, The John Paul II Hospital, 31-202 Kraków, Poland; 4 Department of Cardiac and Vascular Diseases, The John Paul II Hospital, 31-202 Kraków, Poland; 5 Genetic Counselling Outpatient Clinic, The John Paul II Hospital, 31-202 Kraków, Poland; 6 Department of Diagnostic Imaging, University Hospital, 30-688 Kraków, Poland; 7 Chair of Radiology, Jagiellonian University Medical College, 31-501 Kraków, Poland

**Keywords:** Brugada syndrome, atrioventricular conduction, heart rate, genes, next-generation sequencing, family screening, diagnosis, ECG, Holter ECG monitoring, electrocardiogram

## Abstract

Brugada syndrome (BrS) is an arrhythmogenic disorder increasing the risk of syncopal episodes and sudden cardiac death. BrS usually runs through families with reduced penetrance and variable expression. We analyzed the multigenerational family of a patient who died after sudden cardiac arrest with post-mortem diagnosis of BrS. We analyzed clinical history, comprehensive arrhythmic risk, genetic findings, and additional tests, including electrocardiogram (ECG), detailed 24-hour Holter ECG results, and standard echocardiography findings, and followed up the patients in the ambulatory clinic. We analyzed a pedigree of 33 members of four generations of the family (19 male and 14 female patients). In this family, we identified 7 patients with BrS (median Modified Shanghai Score and Sieira model: 4.5 (4–6) and 1 (0–4) points, respectively), including both parents of the deceased patient, and 8 relatives with negative sodium channel blocker drug challenge test. Genetic testing revealed a novel mutation in sodium voltage-gated channel alpha subunit 5 (SCN5A) c.941A>G, (p.Tyr314Cys) inherited from the father of the proband. Patients with BrS were characterized by longer P-wave duration (120 (102–155) vs. 92.5 (88–110) ms, *p* = 0.013) and longer PR intervals (211.3 ±26.3 vs. 161.6 ± 18.9 ms, *p* = 0.001), along with more frequent positive aVR sign, but did not differ in terms of QRS duration or T-wave characteristics in resting ECGs. BrS patients were characterized by lower mean, minimal, and maximal (for all *p* ≤ 0.01) heart rates obtained from Holter ECG monitoring, while there was no difference in arrhythmias among investigated patients. Moreover, visual diurnal variability of ST segment changes and fragmented QRS complexes were observed in patients with BrS in Holter ECG monitoring. There were no major arrhythmic events during median follow-up of 68.7 months of alive BrS patients. These results suggest ECG features which may be associated with a diagnosis of BrS and indicate a novel SCN5A variant in BrS patients. Twelve-lead Holter ECG monitoring, with modified precordial leads placement, may be useful in BrS diagnostics and risk stratification in personalized medicine.

## 1. Introduction

Brugada syndrome (BrS) is an arrhythmogenic disorder increasing the risk of syncopal episodes and sudden cardiac death (SCD), mainly due to ventricular fibrillation (VF) or polymorphic ventricular tachycardia (VT) [1,2,3,4,5,6,7]. The pooled worldwide prevalence of BrS, estimated from population-based electrocardiogram (ECG) studies among adults, is about 5 per 10,000 [8], which classifies it into a group of rare arrhythmogenic disorders predisposing to ventricular tachyarrhythmias [1,8,9].

Most patients (about 80%) are asymptomatic at BrS diagnosis, while about 16% and 3% have experienced syncope or sudden cardiac arrest (SCA), respectively, at this time [10]. BrS manifests mainly during adulthood and the average age of sudden death of BrS patients is about 40 years [11]. Initial ECG descriptions of patients with BrS were published in the second half of the XX century [4,12,13]. According to current clinical practice, it is typical to observe for BrS a spontaneous or induced by sodium channel blocker test (SCBT) or fever, ST-segment elevation ≥2 mm with type 1 BrS morphology in ECG lead(s) V1 and/or V2 positioned in the fourth or higher (third or second) intercostal spaces [14]. ST-segment changes in BrS are dynamic, and in type 1 BrS ECG pattern (coved pattern) are followed by negative T-waves [15,16,17]. BrS, beside the early repolarization syndrome, is included into J-wave syndromes [18].

There were several postulated hypotheses of the pathophysiology of the disease. Both ion channels and/or related proteins in cardiomyocytes may be dysfunctional [19]. The depolarization hypothesis suggesting microscopic changes (e.g., fibrosis) in the right ventricular outflow tract (RVOT), resulting in delayed conduction and depolarization. The repolarization hypothesis claims that the cause is a sodium channel mutation (leading to decrease in a sodium current) and the gradient between endocardial and epicardial action potentials. There is also a theory that the two above-mentioned mechanisms may coexist and BrS is caused by the different embryological origin of the RVOT compared to other cardiac structures [6]. Moreover, currently available evidence does not seem to provide sufficient data to reclassify BrS as a cardiomyopathy [19]. At present, over 40 genes are reported to be involved in BrS, and mutations in the sodium voltage-gated channel α-subunit 5 (SCN5A) gene, which lead to loss of *I*_Na1.5_ channel function, is responsible for 14–34% of BrS cases [5,11,20]. Systematic assessment indicated more than 150 pathogenic/likely pathogenic for BrS SCN5A variants [21]. Mutations in SCN5A are the most common in BrS patients and are inherited as an autosomal-dominant trait with variable expressivity and incomplete penetrance [22].

Assessment of first-degree relatives (FDR) of SCD victims is a very important step towards identification of persons at increased SCD risk and clinical counselling on available preventive or treatment methods [14]. Such approaches were previously reported as useful in clinical practice [23,24,25]. Three-generation pedigree may be helpful in the diagnostic process of family members of patients after SCA [14]. Selecting patients who may benefit most from expanded diagnostics directed towards BrS remains challenging. Comparative evaluation within family members may significantly reduce the number of confounding factors and strengthen the basis for BrS suspicion, leading to the development of a more personalized approach in BrS patients [26].

The aim of this study was to find potential clinical, 12-lead ECG, Holter ECG monitoring, and echocardiographic features distinguishing affected and non-affected by BrS members of the family of a patient with BrS who died after SCA. Moreover, we aimed to assess familial distribution of BrS, genetic findings, and comprehensively evaluate the SCA risk as well as future arrhythmic events in the family members with BrS.

## 2. Material and Methods

### 2.1. Patients

**Figure 1:**
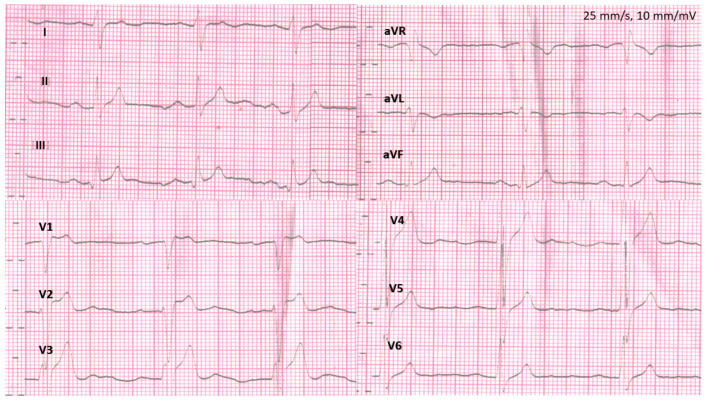
Standard 12-lead electrocardiographic recording of Brugada syndrome patient who experienced sudden cardiac arrest during physical activity. Figure was modified with permission from Matusik PT, et al. [27].

We studied family members of one competitive football player who suffered SCA while playing football. Briefly, the proband had a syncopal episode during physical activity. After this episode, he was referred for a cardiological assessment. In his resting ECG prolonged PQ interval, right bundle branch block and left posterior fascicular block were observed (Figure 1).

The performed echocardiography did not reveal significant structural abnormalities of the heart. Left ventricular end-diastolic diameter was 45 mm, left ventricular end-systolic diameter was 29 mm, right ventricular diameter was 25 mm, and interventricular septum thickness was 11 mm, similarly to inferolateral (posterior) wall thickness. At the same time, left ventricular ejection fraction was 70%, and mild mitral and tricuspid regurgitations as well as small prolapse of anterior leaflet of mitral valve were observed.

**Figure 2:**
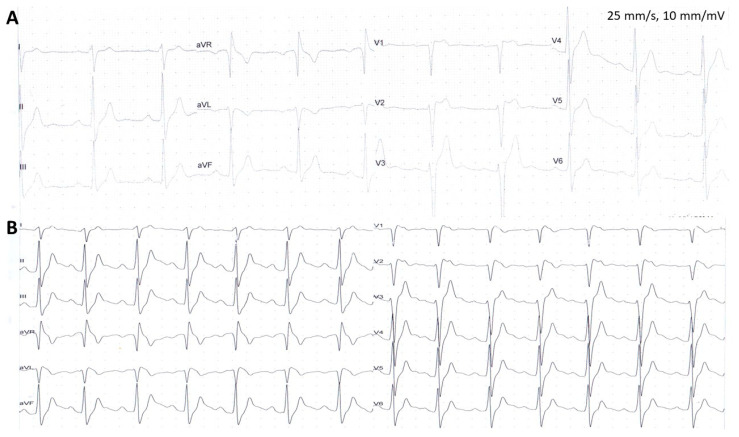
Twelve-lead electrocardiographic (ECG) recordings during exercise testing of Brugada syndrome patient who at later time experienced sudden cardiac arrest during physical activity (Panel (**A**) indicates resting ECG, while Panel (**B**) ECG during recovery phase). Figure was modified with permission from Matusik PT, et al. [27].

Unfortunately, after the syncopal episode, the patient continued his sports activities and about 9 months later, one day after alcohol consumption, suffered SCA during football training. Assessment of the ECG at the time of cardiopulmonary resuscitation (initially performed by bystanders, while later by medical personnel) revealed polymorphic VT and 8 defibrillations were delivered, leading to return of spontaneous circulation. He was admitted to the hospital where performed coronary angiography revealed normal coronary circulation, without atherosclerotic lesions. However, despite intensive treatment (including introduction of neuroprotection), he did not recover consciousness. The subsequent computed tomography scans have shown brain edema with features of intussusception and finally a diagnosis of brain death was established. BrS was diagnosed after death of the patient based on type 1 BrS ECG pattern observed during detailed assessment of the ECG during the recovery phase of the exercise test (Figure 2). The patient and his medical history were described previously in detail [27]. For above-mentioned reasons, we decided to perform wide research on this family, to share our knowledge and clinical experience as well as potentially avoid such dramatic situations in the future.

All first-degree family members of the index case, as well as other FDR of subsequently diagnosed BrS patients, were recommended to undergo cardiological assessment directed towards BrS. Finally, 18 patients were not included in the study, because they were under 18 years of age at initial time of the research or were unable or not willing to perform detailed clinical assessment directed towards BrS.

### 2.2. Brugada Syndrome Diagnosis and Risk Assessment

BrS was diagnosed on the basis of ST-segment elevation with type 1 BrS ECG morphology ≥2 mm in 1 or more leads among the right precordial leads V1 and/or V2 positioned in the fourth or higher (second or third) intercostal space. Patients without spontaneous type 1 BrS ECG pattern underwent SCBT (intravenous administration of ajmaline) to diagnose BrS in accordance with previous and current European Society of Cardiology guidelines [14,28].

We have studied in detail medical documentation of family members referred to the John Paul II Hospital in Kraków with a final diagnosis of BrS and control family members.

We comprehensively assessed the risk of arrhythmic outcomes according to risk factors and models. We calculated the Shanghai Score [29], as well as the Modified Shanghai Score [20], which is a recently reported improved version of the Shanghai Score [29]. The Modified Shanghai Score classifies patients as probable/definite BrS (>3.5 points) and possible BrS (2–3 points) or may be nondiagnostic (<2 points) [20]. Moreover, we performed assessment according to the Sieira model, which takes into account the presence of previous aborted SCD, sinus node dysfunction, previous syncope, inducible electrophysiological study, early familial SCD and spontaneous type 1 BrS ECG pattern [30,31], and distinguishes BrS patients into low- (score 0–1), intermediate- (score 2–4), and high-risk of future arrhythmic events categories (score ≥5) [10].

### 2.3. Electrocardiogram Assessment

**Figure 3:**
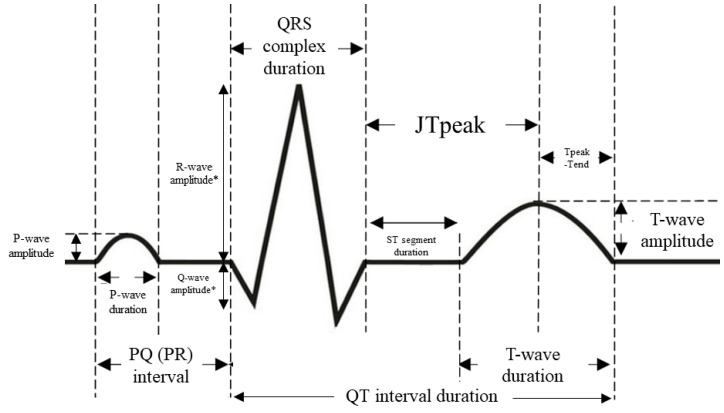
Methodology of 12-lead electrocardiogram assessment. * Measured in lead aVR.

ECG was recorded at a speed of 25 mm/s, with augmentation of 1 cm = 1 mV. ECG recordings were scanned using Plustek OpticSlim 2610 Plus and then were evaluated using an electronic measurement tool with calibration capability. Generally, measurements were made in ECG lead II. We evaluated the duration of the RR intervals, P-waves, PQ (PR) intervals, QRS complexes, QTc intervals, JT peak, T-waves, Tpeak-Tend, as well as voltage of the P-waves and T-waves (Figure 3) [5,32,33]. Prolonged PQ (PR) interval (first-degree atrioventricular block) and QRS complex duration were defined as PQ interval >200 ms and QRS complex duration ≥120 ms, respectively [34]. Moreover, the presence of aVR sign and fragmented QRS complexes were assessed. The aVR sign was defined as R wave ≥ 0.3 mV or R/q ≥ 0.75 in aVR lead [33], while fragmented QRS complexes were defined as ≥3 spikes within the QRS complex in at least one lead V1–V3. The JTpeak and Tpeak-Tend were measured in leads II, V1, and V2. Prolonged Tpeak-Tend interval was defined as Tpeak-Tend interval >100 ms [35].

### 2.4. Holter Electrocardiogram Recordings, Exercise Testing, and Echocardiography

All studied patients except proband before SCA underwent Holter ECG monitoring with the use of Lifecard CF. We have analyzed the available records of 3- or 12-channel Holter ECG monitorings, especially those initially performed and/or those close to BrS diagnosis. We assessed minimum, maximum, and mean heart rates (HR), supraventricular (SVEBs) and ventricular ectopic beats (VEBs), as well as occurrence of potential sustained arrhythmias. Moreover, in some patients we performed 12-lead Holter ECG monitoring with the use of modified precordial leads placement, where leads were placed in the left and right second (V1–V2), third (V3–V4), and fourth (V5–V6) intercostal spaces [36]. Additionally exercise testing results were analyzed when available in medical documentation. Echocardiography studies were acquired based on standard methods.

### 2.5. Implantable Cardioverter-Defibrillator Placement and Parameters during Implantation Procedure

The decision on transvenous implantable cardioverter-defibrillator (ICD) placement in two FDR of the patient who suffered SCA was at the discretion of the treating physicians and the patients. These patients fulfil the criteria for ICD placement according to at least one of previously applicable guidelines [14,37]. Parameters of ICD leads, including impedance, sensing, and pacing thresholds, were assessed during implantation procedure in patients who were referred for ICD placement.

### 2.6. Genetic Testing

A panel of genes associated with BrS was analyzed in the brother of the deceased patient using next generation sequencing (NGS) technology (of note: routinely parents of a proband are assessed, but at the time only the brother was available for testing). The panel included ankyrin 2 (ANK2), calcium voltage-gated channel subunit alpha1 C (CACNA1C), calcium voltage-gated channel auxiliary subunit alpha2delta 1 (CACNA2D1), calcium voltage-gated channel auxiliary subunit beta 2 (CACNB2), caveolin 3 (CAV3), glycerol-3-phosphate dehydrogenase 1 like (GPD1L), hyperpolarization activated cyclic nucleotide gated potassium channel 4 (HCN4), potassium voltage-gated channel subfamily D member 3 (KCND3), potassium voltage-gated channel subfamily E regulatory subunit 3 (KCNE3), potassium voltage-gated channel subfamily H member 2 (KCNH2), potassium inwardly rectifying channel subfamily J member 8 (KCNJ8), RAN guanine nucleotide release factor (RANGRF), sodium voltage-gated channel beta subunit 1 (SCN1B), sodium voltage-gated channel beta subunit 3 (SCN3B), SCN5A, sarcolemma associated protein (SLMAP), and transient receptor potential cation channel subfamily M member 4 (TRPM4). Genetic testing of further family members was mainly targeted towards identified gene mutation and was offered to all alive patients diagnosed with BrS and their available FDR. To date, 5 patients included into the study underwent genetic testing.

### 2.7. Follow-Up, including Assessment of Arrhythmic Outcomes and Device-Related Complications

Patients with BrS were followed-up in the John Paul II Hospital outpatient clinic. Patients with ICD had the device checked regularly. Both device parameters and events in the device memory were assessed. One of the patients was assessed also using telemonitoring.

### 2.8. Statistical Analysis

Variables with normal distribution are shown as means ± standard deviations (SD), while variables with nonnormal distribution are presented as medians and interquartile ranges (IQR, 25th–75th percentile). Age is described by mean values ± SD and medians with IQR. The study group was also described by numbers, percentages, and ranges. Normality of distribution of variables was tested using the Shapiro–Wilk test. T-test and Mann–Whitney U test were used to assess variables with normal and nonnormal distribution, respectively. Analysis of Levene’s test for equality of variances guided *p* value choice in cases of normally distributed data compared with unpaired t-test. Correlations between continuous variables were tested using the Pearson or Spearman rank correlation, as appropriate.

*p* values of <0.05 were considered statistically significant. Statistical analyses were performed with the use of Statistica (version 13; TIBCO Software, Inc., Palo Alto, CA, USA).

## 3. Results

### 3.1. Characteristics of the Patients

**Figure 4:**
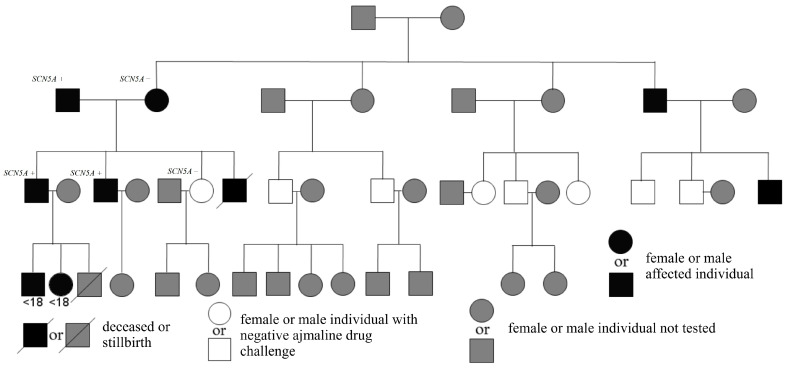
Four-generation family pedigree indicating Brugada syndrome patients with spontaneous and/or drug-induced ST-segment elevation with type 1 Brugada syndrome electrocardiogram morphology.

We analyzed a pedigree of 33 members of four generations of the family (19 male and 14 female patients). The relationships among family members are shown on the genealogical tree (Figure 4). Two of the patients had described positive SCBT performed in the department of pediatric cardiology, at the age of <15 years. Detailed diagnostics in the father’s (father of a deceased BrS patient) family members has not been possible so far. Of his siblings, all died of presumably non-cardiac causes. His parents died at the age of >70 years, while two of his mother’s brothers died at younger age, during physical exertion (the exact details are unknown). Regarding the parents of the mother of a deceased BrS patient, they died at the age of >60 years, due to presumably non-cardiac causes.

**Figure 5:**
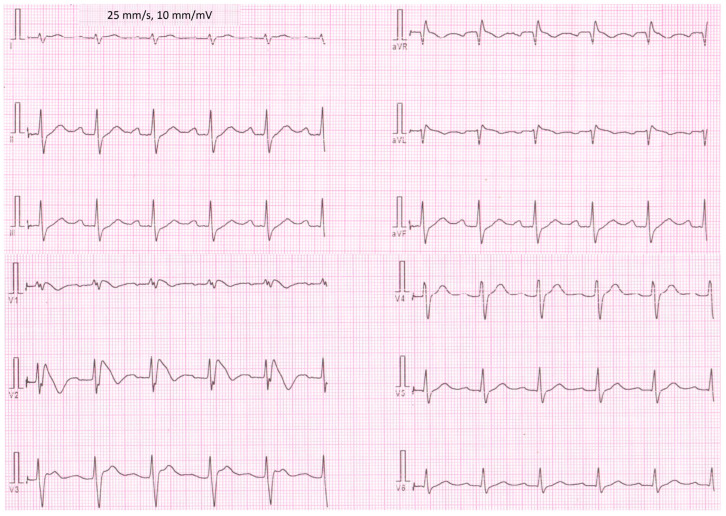
Drug-induced type 1 BrS ECG changes observed in one of the studied patients, both limb and precordial leads are shown.

**Figure 6:**
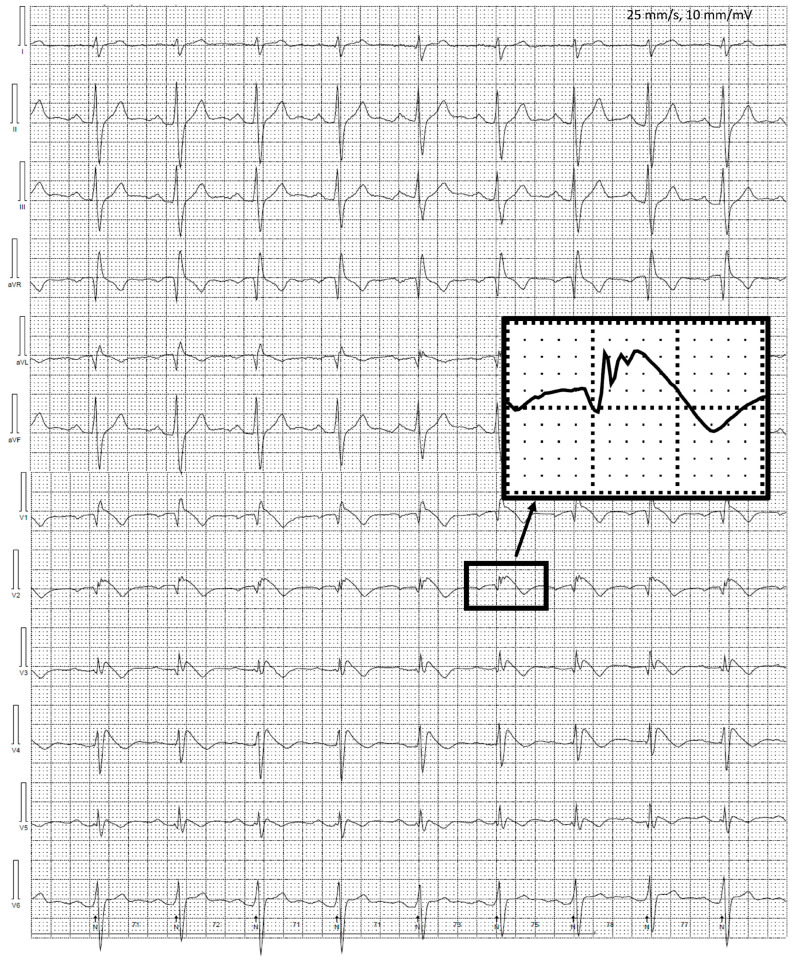
Twelve-lead Holter electrocardiographic recordings with type 1 BrS ECG changes and fragmented QRS complexes in one of the patients with Brugada syndrome. The precordial leads were modified: leads V1 and V2 were located in the second intercostal space, leads V3 and V4 in the third intercostal space, and leads V5 and V6 in the fourth intercostal space.

**Figure 7:**
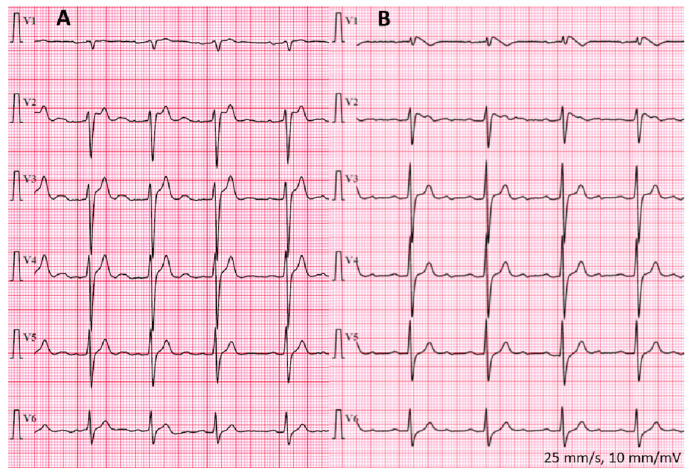
Precordial leads of standard 12-lead electrocardiograms (ECG) from studied Brugada syndrome (BrS) patients (Panel (**A**): features of type 2 BrS ECG changes, Panel (**B**): spontaneous type 1 BrS ECG changes).

Detailed investigation was possible in 15 patients included in the study in mean age (at first detailed assessment or performance of SCBT, Figure 5) of 36.0 ± 13.5 (22–65) years, median 32 (25–40) years. Females consisted of 26.7% (*n* = 4). Among the studied patients, 7 had type 1 BrS ECG pattern, which in 3 patients was spontaneous (including 1 patient in whom it was observed during recovery phase of exercise test), while in the remaining 4 patients was induced during SCBT (intravenous administration of ajmaline, Figure 5, Figure 6 and Figure 7). The median Modified Shanghai Score and Sieira model in patients with type 1 BrS ECG pattern were 4.5 (4–6) and 1 (0–4) points, respectively. In this subgroup of patients, four persons (57.1%) also exhibited type 2 BrS ECG pattern.

Relatives who were below 18 years old during the recruitment phase of this study and had positive for Brugada syndrome SCBT performed at another institution (department of pediatric cardiology) are indicated by “<18”.

All BrS patients had modified Shanghai scoring system score classifying them as probable/definite BrS. Sieira model assessment indicated 1 patient (14.3%) as having high-risk, 2 patients (28.6%) as being in the intermediate-risk category, while 4 patients (57.1%) were in the low-risk of future events category. Eight patients did not reveal type 1 BrS ECG pattern during SCBT. In these patients, the median Modified Shanghai Score and Sieira model were 2.0 (2.0–2.0) and 0 (0–0) points, respectively. A comparison of the basic clinical characteristics and risk factors observed in patients with BrS and those with negative SCBT are presented in Table 1.

The age and sex of patients with BrS did not differ from patients without type 1 BrS ECG pattern induced during SCBT (43.0 ± 16.7 vs. 29.9 ± 6.0 years; *p* = 0.16 and 14.3% women vs. 37.5% women; *p* = 0.57).

### 3.2. Electrocardiogram Assessment

Patients with BrS compared to the remainder patients were characterized by more prevalent aVR sign (Table 1), longer P-wave duration (120 (102–155) vs. 92.5 (88–110) ms, *p* = 0.013) and PQ intervals (211.3 ± 26.3 vs. 161.6 ± 18.9 ms, *p* = 0.001), as well as more frequent presence of first-degree AV block in resting ECG (Table 2).

We observed no difference in RR interval duration, QRS complex duration, and QTc interval duration measured in lead II between patients with and without BrS. Moreover, JTpeak (*p* = 0.42, *p* = 0.54, *p* = 0.64) and Tpeak-Tend (*p* = 0.91, *p* = 1.0, *p* = 0.63) measured in lead II, V1, and V2, did not differ between patients with type 1 BrS ECG pattern and the remainder persons (Table 2).

A trend for shorter T-wave duration and lower T-wave voltage was observed among patients with BrS (165 vs. 193 ms, *p* = 0.093 and 0.20 vs. 0.36 mV, *p* = 0.083), compared to patients without BrS. Detailed comparative data are presented in Table 2.

PQ interval duration correlated with duration of P-wave (r = 0.85, *p* < 0.001), QRS (R = 0.57, *p* = 0.026) and T-wave (r = −0.54, *p* = 0.036), as well as predictive risk models (Shanghai Score: R= 0.89, *p* < 0.001; Modified Shanghai Score: R = 0.78, *p* < 0.001; Sieira model: R = 0.74, *p* = 0.001).

### 3.3. Twenty-Four-Hour Holter Electrocardiogram Recordings, Exercise Testing, and Echocardiography

Adult BrS patients compared to the patients without BrS were characterized by lower mean, minimum, and maximum HR obtained from Holter ECG monitoring (65.0 ± 7.0 beats per minute [bpm] vs. 75.1 ± 6.9 bpm, *p* = 0.019; 48.2 ± 6.1 vs. 54.4 ± 4.6 bpm, *p* = 0.049 and 97.3 ± 8.5 vs. 121.1 ± 9.4 bpm, *p* < 0.001, respectively). Moreover, HR calculated from Holter ECG monitoring correlated with PQ interval duration (mean HR: *p* = 0.033, r = −0.57; minimum HR: *p* = 0.038, r = −0.56; maximum HR: *p* = 0.001, r = −0.78).

On the other hand, we did not observe any difference regarding SVEBs (*p* = 0.26) or VEBs (*p* = 0.74) between studied adult patients with and without BrS. There was no nonsustained or sustained VT in compared between groups Holter ECG recordings.

**Figure 8:**
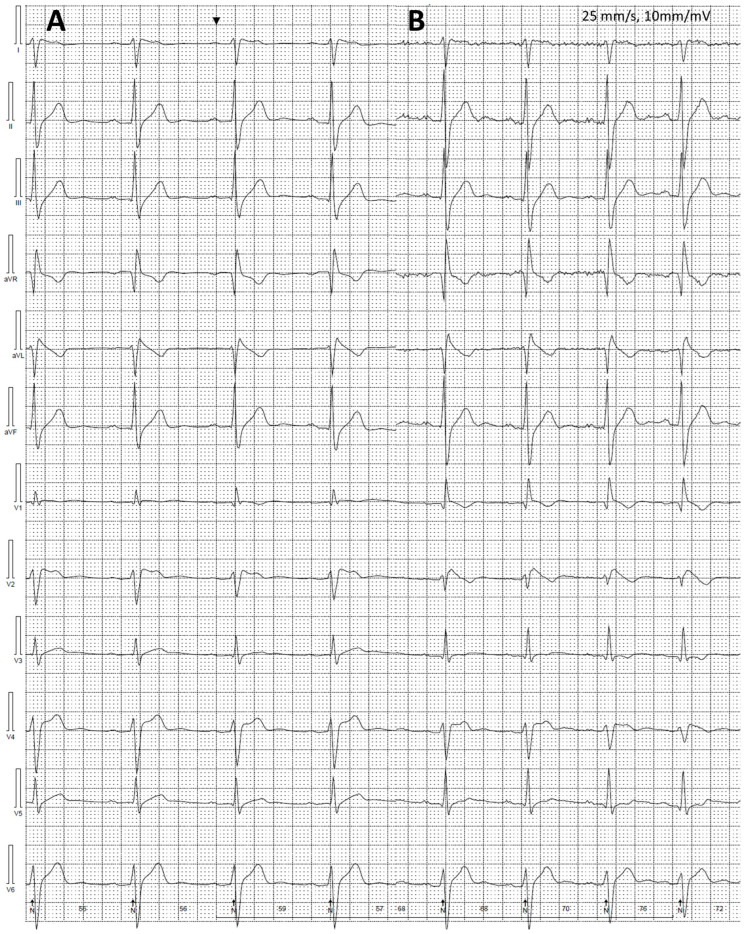
Variability of electrocardiographic (ECG) recordings in a studied patient with Brugada syndrome. The precordial leads were modified: leads V1 and V2 were located in the second intercostal space, leads V3 and V4 in the third intercostal space, and leads V5 and V6 in the fourth intercostal space. Change of ECG recording are especially notable in lead V2: Panel (**A**) indicates features of type 2 BrS ECG changes (9:23 PM), while Panel (**B**) indicates type 1 BrS ECG changes (7:34 AM). Both recordings are from the same Holter ECG monitoring.

We have shown visual diurnal variability of the ST segment, including occurrence of type 1 BrS ECG patterns (Figure 8, please note the similarities in conduction abnormalities with the deceased brother) and fragmented QRS complexes (Figure 6) among patients with BrS in 12-lead Holter ECG monitoring.

There was no sustained arrhythmia induced during exercise testing in our cohort. Moreover, investigated patients with (*n* = 7) or without (*n* = 6) BrS did not differ in regard to left ventricular (LV) ejection fraction (*p* = 0.61), LV end-diastolic and end-systolic diameter (*p* = 0.55 and *p* = 0.92, respectively), left atrial diameter (*p* = 0.24), or interventricular septum thickness (*p* = 0.24), Table 2.

### 3.4. Genetic Testing Results

To date, we have performed genetic testing in five adult patients. The brother of the deceased patient was diagnosed with a heterozygous variant of the SCN5A c.941A>G, p.Tyr314Cys gene. This novel variant was not described in ClinVar and gnomAD databases. Targeted testing for the SCN5A c.941A>G, p.Tyr314Cys mutation was also positive in his brother and the father.

The results of the targeted testing in the mother and in the sister with negative SCBT were negative. The gene was analyzed in all FDR of the deceased patient. There were no significant differences in ECG and echocardiographic parameters between patients with and without the SCN5A c.941A>G, p.Tyr314Cys mutation.

### 3.5. Follow-Up

There were no major arrhythmic events and complications related to transvenous ICD during a median follow-up of 68.7 months in six alive patients diagnosed with BrS; none of them experienced aborted SCD. However, nonsustained VT was detected by an ICD (ca. 10 consecutive beats at an average rate of 200 bpm) of one of the BrS patients.

## 4. Discussion

In this study, we have found 12-lead ECG and Holter ECG monitoring features distinguishing affected and non-affected by BrS members of the family of a patient with BrS who died after SCA during exercise. On the other hand, standard echocardiographic parameters did not differ between these groups of patients, indicating that ECG parameters may provide potential clue for clinical counselling and management. Moreover, we assessed familial distribution of BrS, comprehensively evaluated SCA risk, and identified a novel mutation in the SCN5A gene in the family members with BrS.

### 4.1. Characteristics of the Patients

The majority of BrS patients studied by us did not experience severe symptoms at BrS diagnosis, similarly to BrS patients studied by Probst et al. [10]. Moreover, it should be noted that syncope or SCA may be the first manifestation of the disease [38], as in our index case. In most BrS cases, arrhythmic events occur during sleep, resting conditions, or in situations of elevated body temperature [11,28,39], which may also induce BrS ECG changes [26,40,41]. Unexpectedly, during initial evaluation, our proband experienced SCA and earlier syncopal episode during physical activity, which is more typical for arrhythmogenic right ventricular cardiomyopathy (ARVC) [42]. However, it should be noted that there were cases of SCA (VF while running [43]) or sudden death (SCN5A mutation carrier [44]) of patients with BrS during or after exercise described. As previously reported by us in detail, the presented patients highlight how challenging the determination of the etiology and management of syncopal and/or presyncopal episodes [45,46] can be, and that unusual triggers, such as physical exercise, may also contribute to SCD among patients with BrS. Also of significance, the dominant subtype of ventricular arrhythmia may help to guide clinical diagnostics (e.g., in the case of ARVC the predominant ventricular arrhythmia is monomorphic VT) [28].

As analyzed by Probst et al. [10] in a large database of patients with BrS, the Shanghai Score (evaluated in 1613 patients) was <3 in 25.3% of patients compared to 42.9% of BrS patients studied by us. However, all BrS patients in our study had a more recent, modified Shanghai scoring system score classifying them as probable/definite BrS. This score highlights the need for the presence of other criteria in the absence of a spontaneous type 1 BrS ECG pattern and seems to be useful in clinical diagnostics of BrS [47]. Another diagnostic insight from our study is related to the fact that we report a family of initially two unrelated patients with BrS who have offspring. This example shows that BrS diagnosis should be considered in both parents of the index case, even when one of them has already been diagnosed with BrS. Importantly, resting ECG in patients with BrS often does not show type 1 BrS ECG pattern, thus SCBT may reveal characteristic changes. However, we must keep in mind that the choice of a drug used during SCBT matters and in BrS patients’ discordant results during SCBT using flecainide and ajmaline have been reported [47,48].

According to current European Society of Cardiology guidelines, BrS diagnostics should also be carried out in children [28]. ECG and high precordial lead ECGs should generally start in children at least 10 years old, while SCBT should usually start above 16 years, unless clinically indicated [28]. In two children from the investigated family, the ajmaline test was performed at <15 years old, before these ESC guidelines were published. There are data suggesting that ajmaline challenge in children ≤12 years old may be associated with a higher risk of sustained ventricular arrhythmias (VF or sustained VT) [49]. Moreover, a SCBT performed before puberty may be false negative [50].

The important parameters that increase the risk of arrhythmic events in BrS and are included in the Sieira model are previous aborted SCD, sinus node dysfunction, previous syncope or inducible electrophysiological study, early familial SCD, and spontaneous type 1 BrS ECG pattern [30]. In our cohort Sieira model, assessment indicated 1 patient (14.3%) as having high-risk, 2 patients (28.6%) as being in the intermediate-risk category, while 4 patients (57.1%) were in the low-risk of future events category. At the same time in the study by Probst et al. (the Sieira risk score was assessed in 461 patients), there was relatively lower percentage of high- (5%) and low-risk (45.8%) patients [10]. However, it should be noted that the Sieira model does not allow precise stratification of the risk of arrhythmic events in intermediate-risk patients, for whom management remains the most difficult, while the use of the Shanghai Score did not improve SCA risk stratification [10].

### 4.2. Twelve-Lead ECG

Patients with BrS were characterized by longer PR intervals and more frequent first-degree AV block in the resting ECGs. It was reported that the presence of first-degree AV block is associated with the presence of SCN5A mutation [34]. Our study showing a high percentage of first-degree AV block in patients with SCN5A mutation is in line with these findings. Moreover, these results should be considered in light of findings of a meta-analysis, which has shown that first-degree AV block is associated with more frequent major arrhythmic events in BrS patients [51].

Importantly, some of the ECG changes observed in BrS patients may occur physiologically in athletes, as training-related ECG alterations, including sinus bradycardia (≥30 bpm), first-degree AV block, incomplete right bundle branch block, and ST or J-point elevation [52]. Furthermore, convex ST-segment elevation together with T-wave inversion (in leads V1–V4) may be considered a normal variant in black/African athletes [52]. The Corrado index and β angle assessment may be helpful in distinguishing BrS ECG changes from ECG alterations observed in athletes [16].

In the analyzed group of patients, we observed a higher occurrence of aVR sign in the resting ECG in patients with BrS compared to patients with negative SCBT, expanding the findings of the previous study which reported aVR sign only in BrS patients with spontaneous type 1 BrS ECG pattern [53]. It should be underlined that aVR sign is associated with a worse prognosis (arrhythmic events) in patients with BrS [33,54].

### 4.3. Holter ECG Monitoring and Echocardiography

BrS patients within one family are characterized by lower HR. This is in line with previous observations in patients in Thailand indicating a trend towards lower average HR in BrS compared to controls and asymptomatic patients with Brugada ECG [55]. Interestingly, this trend was mostly driven by a lower HR during the day, as during the night the average HR in patients with BrS was higher [55].

Moreover, we noticed visual diurnal variability of ST segment changes, including the presence of type 1 BrS ECG pattern in some patients with BrS, suggesting a utility from prolonged 12-lead Holter ECG monitoring with modified precordial leads in BrS diagnostics and risk stratification. This is in accordance with Shimeno et al., who have found multichannel Holter ECG recording in the third intercostal space as useful and more sensitive in BrS diagnostics compared to repeated 12-lead ECGs or Holter monitoring with standard leads placement [56]. Our approach of modified precordial leads placement in 12-lead Holter ECG monitoring was previously described as capable to identify 34% of patients initially diagnosed as “drug-induced BrS” as patients with spontaneous BrS ECG pattern [36]. These dynamic changes in ECG patterns and ST elevation are other ECG features, which differentiate BrS from ARVC, beside the potential spread of T-wave inversions [15].

Fragmented QRS complexes, among family members investigated by us, were observed in 28.6% of BrS patients. The occurrence of fragmented QRS complexes depends on used definition, methodology of ECG recordings, and population studied. In the PRogrammed ELectrical stimUlation preDictive valuE (PRELUDE) registry which included patients with spontaneous or drug-induced type 1 BrS ECG pattern without a history of cardiac arrest at study enrollment, QRS fragmentation (defined as ≥2 spikes within QRS complex in leads V1-3) was observed in 8.1% of patients and was a significant predictor of arrhythmias [57]. In another study, Morita et al., who defined fragmented QRS complexes as ≥4 spikes within QRS complex in 1 lead or ≥8 spikes in 3 leads of V1–V3, the percentage of fragmented QRS complexes reached 43% of BrS patients and was more frequent in a group of patients with a history of VF, compared to patients with syncope or asymptomatic patients [58]. Identification of fragmented QRS complexes may play a role in a more personalized approach to SCD risk stratification.

BrS is usually presented as a disease with anatomically normal cardiac structures, which was also observed in our patients studied with standard echocardiographic evaluation. However, advanced imaging techniques were reported to reveal subtle changes in the right ventricle. Currently, increasing evidence suggests small-scale structural changes in the heart, mainly in cardiac MRI examination [59,60,61]. Moreover, overlapping phenotypes of BrS and ARVC have been suggested and could result from loss of expression of desmosomal proteins [15].

### 4.4. Genetic Findings

In BrS, the pathogenic variant is inherited from the affected parent or may occur de novo. In most cases, it is inherited in an autosomal dominant manner. SCN5A variants are main contributors to BrS [62]. Importantly, the SCN5A c.941A>G, p.Tyr314Cys mutation described by us is the first case described in the literature, among patients with BrS. Moreover, it should be noted that functionally proven loss-of-function SCN5A mutation carriers are characterized by enhanced conduction abnormalities and worse prognosis [63]. Thus, such assessment could be valuable in our patients.

Each child of a person with autosomal dominant mutation implicated into BrS has a 50% chance of inheriting the mutation, but the risk of developing BrS may be lower due to reduced penetrance and other influencing factors [11]. In the analyzed family, in which both parents were diagnosed with BrS, the risk of their children developing the disease may be higher. Among our patients, some family members may also have two pathogenic variants which may increase the chances of developing the disease in offspring. There were no significant differences in ECG and echocardiographic parameters between patients with and without the found novel variant in the SCN5A gene. However, interestingly, van Hoorn et al. [60], in a larger group of patients with BrS investigated with ECG and cardiac MRI, found that BrS patients with SCN5A mutations had decreased HR and increased PR and QRS durations compared to SCN5A-mutation-negative patients. Moreover, they found that these patients were characterized by increased end-diastolic and end-systolic right ventricular and end-systolic LV volumes and decreased LV ejection fraction, compared to SCN5A-mutation-negative patients and age/sex-matched healthy volunteers [60].

### 4.5. Limitations of the Study

Several limitations of the study should be acknowledged. The study group is relatively small, especially in the case of comparisons between patients with and without SCN5A mutation, but sufficient to observe clinically valuable differences between the main studied groups in terms of classical ECG and Holter ECG parameters. Genetic testing is still underway in the reported family (we plan to further investigate the mother and at least maternal side of the family). However, due to the importance of the obtained findings, we consider it very important to publish our preliminary results before final diagnostics in the family.

### 4.6. Future Perspectives

Average, minimal, and maximal HR and their derivatives at different times of the day, as well as 12-lead ECG parameters, should be tested in large prospective studies, preferably combined with clinical data, to assess their predictive value. Detailed approaches to clinical evaluation, including multiparametric risk scores, described variants implicated into BrS, and/or biomarkers, should be assessed to better understand the mechanisms and potentially improve diagnostics and risk stratification as well as to identify gaps in the management of BrS patients, as in other cardiovascular diseases [64,65,66,67,68,69,70]. Moreover, further, long-term studies on subcutaneous ICD, especially in light of advances in this field, in BrS patients should be performed [71,72,73].

## 5. Conclusions

BrS is an inherited cardiac disease and implicates intensive family members screening. Our results show 12-lead ECG features which may be associated with a diagnosis of BrS. Genetic testing resulted in the identification of a novel variant of the SCN5A gene. The affected persons seem not to differ significantly from those free from the disease according to age, number of ventricular extrasystolic beats in Holter ECG monitoring, as well as left ventricular dimensions or function assessed by standard transthoracic echocardiography. Twelve-lead Holter ECG monitoring, with modified precordial leads placement, may be useful in BrS diagnostics and risk stratification in personalized medicine.

## Figures and Tables

**Table 1:** Basic characteristics of patients with and without (and with negative sodium channel blocker test) type 1 Brugada syndrome ECG changes. Data were also gathered and assessed during follow-up.

Variable	Type 1 BrS ECG Pattern (*n* = 7)	Without Type 1 BrS ECG Pattern (*n* = 8)	*p*-Value
Age (years)	43.0 ± 16.7	29.9 ± 6.0	0.16
Male sex (%)	6 (85.7%)	5 (62.5%)	0.57
Type 2 BrS ECG changes	4 (57.1%)	0 (0%)	0.026
Syncope ^$^	2 (28.6%)	0 (0%)	0.20
Syncope or presyncope associated with physical activity ^$^	2 (28.6%)	0 (0%)	0.20
Early (<45 years) SCD or aborted SCD in FDR	4 (57.1%)	1 (12.5%)	0.10
Relation to index-case			
FDR	4 (66.7%)	1 (12.5%)	0.028
2nd-degree relative	1 (16.7%)	0 (0%)	
3rd-degree relative	1 (16.7%)	7 (87.5%)	
Scores for diagnosis and/or arrhythmic outcomes risk assessment			
Shanghai Score #	4.0 (2.5–6.0)	2.0 (2.0–2.0)	0.002
Modified Shanghai Score #	4.5 (4.0–6.0)	2.0 (2.0–2.0)	<0.001
Sieira model *	1.0 (0–4.0)	0 (0–0)	0.021
Positive aVR sign	5 (71.4%)	1 (12.5%)	0.03
Fragmented QRS complexes **	2 (28.6%)	0 (0%)	0.20

Values are shown as mean ± standard deviation, median (interquartile range) or number (percentage). ECG—electrocardiogram, FDR—first-degree relative, PA—physical activity, SCBT—a sodium channel blocker test, SCD—sudden cardiac death, * None of the patients had EPS performed, ** Observed in one patient in 12-lead ECG, while in one in 12-lead Holter ECG. # Taking into account that diagnosed mutation is considered probably pathogenic. ^$^ In 1 patient syncope occurred during PA, in another one after injury of the hand, while in one of the patients presyncope after PA was noted.

**Table 2:** Twelve-lead electrocardiography, 24-h Holter electrocardiography monitoring, and echocardiography results in family members with type 1 BrS ECG pattern and the remainder patients.

Variable	Type 1 BrS ECG Pattern (*n* = 7)	Without Type 1 BrS ECG Pattern (*n* = 8)	*p*-Value
Twelve-lead ECG analysis			
RR interval duration [ms]	900 (811–1066)	830.5 (754–929.5)	0.27
P-wave duration [ms]	120 (102–155)	92.5 (88–110)	0.013
P-wave voltage [mV]	0.13 (0.05–0.14)	0.09 (0.08–0.10)	0.45
PQ (PR) interval duration [ms]	211.3 ± 26.3	161.6 ± 18.9	0.001
Prolonged PQ interval duration, *n* [%]	4 (57%)	0 (0%)	0.026
QRS complex duration [ms]	125 (87–128)	89 (86–98.5)	0.18
Prolonged QRS complex duration, *n* [%]	4 (57%)	0 (0%)	0.026
QTc interval duration [ms]	401 (387–403)	389 (376–427)	0.52
JTpeak interval in V1 [ms]	172.9 ± 29.7	183.1 ± 32.9	0.54
JTpeak interval in V2 [ms]	165 ± 24.2	170.6 ± 21.5	0.64
T-wave duration [ms]	165 (165–195)	193 (178–204)	0.093
T-wave voltage [mV]	0.20 (0.20–0.23)	0.36 (0.21–0.53)	0.083
Tpeak-Tend interval in V1 [ms]	85 ± 17.2	85 ± 16.6	1.0
Tpeak-Tend interval in V2 [ms]	105.8 ± 9.1	101.4 ± 22.7	0.63
Prolonged Tpeak-Tend interval in V1, *n* [%]	1 (17%)	2 (33%)	0.55
Prolonged Tpeak-Tend interval in V2, *n* [%]	4 (57%)	6 (75%)	0.43
Holter ECG monitoring analysis ^#^			
Mean heart rate [bpm]	65.0 ± 7.0	75.1 ± 6.9	0.019
Minimal heart rate [bpm]	48.2 ± 6.1	54.4 ± 4.6	0.049
Maximal heart rate [bpm]	97.3 ± 8.5	121.1 ± 9.4	<0.001
Supraventricular extrasystolic beats [*n*]	5.5 (2.0–12.0)	0.5 (0.0–5.5)	0.26
Ventricular extrasystolic beats [*n*]	1.0 (0.0–7.0)	3.0 (0.5–18.5)	0.74
Echocardiography study results ^##^			
Left ventricular ejection fraction [%]	65.0 (63–70)	65.0 (62–66)	0.61
Left ventricular end-diastolic diameter [mm]	48.9 ± 3.4	47.8 ± 2.4	0.55
Left ventricular end-systolic diameter [mm]	31.8 ± 6.3	31.4 ± 3.6	0.92
Interventricular septum thickness [mm]	9.9 ± 1.1	9.7 ± 1.8	0.24
Posterior wall thickness [mm]	10.0 (10.0–11.0)	10.5 (9.0–11.0)	0.77
Left atrial diameter [mm]	37.3 ± 4.3	34.5 ± 3.4	0.24
Ascending aorta diameter [mm]	29.8 ± 4.0	28.3 ± 3.5	0.51

Values are shown as mean ± standard deviation, median (interquartile range), or number (percentage). ^#^ Holter ECG results for a patient after SCA were available only after SCA, therefore were not included into analysis. ^##^ In a patient after SCA included echocardiography results were obtained >6 months before SCA; echocardiography results were available for 6 persons without type 1 BrS ECG changes.

## Data Availability

The data presented in this study are available on a reasonable request from the corresponding author.
